# Fear of COVID-19, Stress, and Anxiety in University Undergraduate Students: A Predictive Model for Depression

**DOI:** 10.3389/fpsyg.2020.591797

**Published:** 2020-11-05

**Authors:** Antonio J. Rodríguez-Hidalgo, Yisela Pantaleón, Irene Dios, Daniel Falla

**Affiliations:** ^1^Department of Psychology, Cátedra de Cooperación al Desarrollo, University of Cordoba, Cordoba, Spain; ^2^Department of Education, University Laica Eloy Alfaro of Manabí, Manta, Ecuador; ^3^Department of Psychology, University of Cordoba, Cordoba, Spain

**Keywords:** COVID-19, depression, fear, stress, anxiety, undergraduate students

## Abstract

Depression is a disabling illness which increases the risk of suicide. The Corona Virus Disease 2019 (COVID-19) pandemic has led to a rise in fear, anxiety, stress, and depression among the population: of these, university undergraduates from countries severely affected by COVID-19 are some of the most vulnerable of all, as they face strict lockdown measures and have fewer resources to cope with it. The aim of this study was to analyze the levels of fear of COVID-19, stress, anxiety, and depression during lockdown among undergraduates from Ecuador, and to test these possible predictors of depression using a model taken from our study of the scientific literature. A total of 640 undergraduates (72% women) between 18 and 47 years old (*M* = 21.69; *S.D* = 4.093) were surveyed. The resulting mean levels found for stress, anxiety, and depression were above levels considered non-pathological. Women showed higher levels of fear of COVID-19 than men. The statistical prediction for depression showed a good fit. This depression could be related: both directly and positively by fear of COVID-19 and stress, and indirectly, as a result of these two factors, positively mediated by anxiety. Our study concludes by highlighting the important role that the complex relationships between fear, stress, and anxiety can play in the development of depression symptoms and how they can be taken into account in programs aimed at preventing and alleviating this disorder. We propose some general measures for reducing fear of COVID-19 and stress and suggest that specific programs be designed to control and overcome anxiety among undergraduates.

## Introduction

Depression is one of the main factors that generates disability in populations in modern societies (Dong et al., 2020; Nuggerud-Galeas et al., 2020). Having experienced epidemics or natural disasters increases long-term levels of depression in populations (Mak et al., 2009; Lee et al., 2018; Morganstein and Ursano, 2020) and may also increase their future suicide rates (Cheung et al., 2008). Experiencing more upsetting events in life and finding it difficult to cope with them are also predictors of anxiety, stress, and depression (Zou et al., 2018). At present, the world is facing a critical situation caused by the Severe Acute Respiratory Syndrome (SARS)-CoV-2 virus, and this has contributed hugely toward increasing levels of depression in the population in different countries. The situation of the population in some countries which have been severely affected by the epidemic and have little ability to cope, as is occurring in several Latin American countries, is particularly alarming. In the current epidemic crisis, studying the causes of depression in vulnerable contexts can be of great strategic value to help alleviate this illness now and prevent it in the future.

### COVID-19 in Latin America

In Latin American countries, the first case was reported in Brazil on February 25, 2020 (Rodriguez-Morales et al., 2020), and after that, the disease expanded rapidly throughout this vast region. In Ecuador, there are now over 65,000 people infected, and this country has become one of the worst affected in all Latin America (Muñoz, 2020). Like other countries with limited financial resources and deficient healthcare facilities, Ecuador has had serious difficulties in identifying possible cases of infection, stopping its spread, and treating patients (Hoffman and Silverberg, 2018; Kapata et al., 2020). This critical situation, set in a context of great vulnerability, can lead to a feeling of extreme helplessness among the population, which particularly affects mental health (Llibre-Guerra et al., 2020). Thus, recent studies in Latin American countries have found that health care workers have symptoms of anxiety and distress (Chen et al., 2020b; Yáñez et al., 2020; Zhang et al., 2020); while, in other studies, almost half reported symptoms of depression (Guiroy et al., 2020). However, despite the fact that some studies have reported that these symptoms occur to a greater extent in the population between 18 and 28 years of age (Cortés-Álvarez et al., 2020), hardly any studies have been carried out in university students.

### The COVID-19 Pandemic and Depression

The SARS-CoV-2 virus particularly affects the respiratory system and is highly infectious, with a long incubation period. The World Health Organization (WHO) has named the disease Corona Virus Disease 2019 (COVID-19; Wu et al., 2020). It was first discovered in the city of Wuhan (China) in 2019, but its remarkable ability to spread and its rapid expansion around the world has led the WHO to consider it a pandemic (Rothan and Byrareddy, 2020). This pathogen has now become one of the worst health, social, and economic problems worldwide in recent years (Nicola et al., 2020). A number of studies have shown the impact that COVID-19 can have and its effects on people’s well-being, due to its ability to produce a full-scale mental health crisis, especially in countries with a large number of people affected by the disease (Fiorillo and Gorwood, 2020). A number of studies have already begun to look at psychological disorders such as depression in populations affected by COVID-19 (e.g., Duan and Zhu, 2020; Gao et al., 2020; Huang and Zhao, 2020). Depression is a disorder made up of symptoms associated with low morale—despair, sadness, self-depreciation, and worthlessness—leading to reduced self-esteem and lack of interest in life. The disorder is closely linked to a lower probability of achieving significant life goals for those who suffer from it, with worsening health and with suicide attempts (Antúnez and Vinet, 2012; Roh et al., 2020; Siegrist and Wege, 2020; Zhuo et al., 2020). In order to prevent and alleviate depression during the current crisis and in the post-COVID-19 world, we need to look into the factors associated with this disorder.

### Fear of COVID-19, Stress, Anxiety, and Depression

The pandemic has forced many governments to bring in strict laws to stop it from spreading (Adhikari et al., 2020). The governments of the worst affected countries, in terms of number of infections, patients, and mortality levels, such as China, Italy, Spain, and Ecuador, have decreed long periods of self-isolation and/or lockdown, in which citizens have had to stay home. This has seriously affected the living conditions of their populations, and it has been especially detrimental in countries with fewer resources, such as those in the Latin American region. Certain aspects of the disease, such as the uncertainty about how it is spread, its evolution or about the immunity of patients who have been infected, or the absence of a vaccine to counter the disease, have led to an increased feeling of fear among the population (Orellana and Orellana, 2020; Ornell et al., 2020; Rodríguez-Rey et al., 2020).

These fears, generated by the perception of threatening stimuli, have already been seen in previous epidemics, such as those caused by SARS (Reynolds et al., 2008) or Middle East Respiratory Syndrome-Coronavirus (MERS-CoV; Bukhari et al., 2016). Given the severe global threat and impact that the COVID-19 pandemic has produced on different aspects of human survival, health, well-being, and development, Ahorsu et al. (2020) designed a scale to measure the fear of this pathogen based on the existing scientific literature: the Fear of COVID-19 Scale (FCV-19S). This scale has been used in a wide range of countries, such as Iran (Alyami et al., 2020), Bangladesh (Sakib et al., 2020), Italy (Soraci et al., 2020), Turkey (Satici et al., 2020), Russia and Belarus (Reznik et al., 2020), Israel (Tzur Bitan et al., 2020), Peru (Huarcaya-Victoria et al., 2020), and Paraguay (Barrios et al., 2020).

Most of these studies also detected a link between fear of COVID-19 and anxiety (Mertens et al., 2020) and, to a lesser extent, depression, using both the Hospital Anxiety and Depression Scale (HADS; Ahorsu et al., 2020; Alyami et al., 2020) and the Depression and Anxiety Stress Scale (DASS-21). It has recently been observed that fear of COVID-19 is associated more with anxiety and stress and to a lesser extent with depression (Tzur Bitan et al., 2020). Nevertheless, despite the fact that there seems to be a lesser association between fear and depression, cases of suicide have been reported in the population due to fear of COVID-19 (Mamun and Griffiths, 2020).

In addition, the high daily rates of new cases and deaths together with the bombardment of information to which citizens are submitted through the media can influence the development of mood disorders (Duan and Zhu, 2020; Gao et al., 2020). Thus, from the early stages of the pandemic, Chinese researchers found moderate and severe symptoms of anxiety, stress, and depression in the Chinese population (Huang and Zhao, 2020).

The relationships between stress, anxiety, and depression have long been documented in the scientific literature. The theoretical models, supported by scientific evidence, link socioenvironmental stress with internal biological processes that drive the pathogenesis of depression (e.g., Slavich and Irwin, 2014; Park et al., 2019). Longitudinal studies in young people also suggest that stress predicts depression (e.g., Agoston and Rudolph, 2011). We know that in highly stressful situations, there is a close link between anxiety and depression (Díaz et al., 2012), for instance, in people suffering from post-traumatic stress disorder, who often show high levels of fear and anxiety (Forbes et al., 2010). Anxiety and depression are also known to be positively related (Jansson-Fröjmark and Lindblom, 2008) and both function as predictors of each other (Jacobson and Newman, 2017; Hovenkamp-Hermelink et al., 2019).

In the current crisis caused by the pandemic, the emerging literature is beginning to reveal certain differences based on gender and age. Women and younger people show higher levels of depression, anxiety, stress, and fear of COVID-19 (Huang and Zhao, 2020; Sandín et al., 2020). However, most of these studies were carried out in samples of health workers (Pappa et al., 2020), and much less is known about young people. Undergraduate students at university have been observed to be more fearful of COVID-19 than graduates (Reznik et al., 2020). In addition, according to some studies, the symptoms of anxiety and depression among these students are increasing due to social distancing and lockdown laws (Chen et al., 2020a; Mazza et al., 2020; Santini et al., 2020).

### The Present Study

After reviewing the emerging literature on the critical situation of global pandemic caused by COVID-19, it is clear that more research is needed on the possible predictors of depression. The focus of this study is university undergraduate students, who, in particular, seem to be a highly vulnerable population. We conducted the research in Ecuador, in order to learn more about the relationship between these psycho-social factors in a country potentially affected by high levels of stress and fear, with extremely restrictive measures of social distancing and lockdown in force, with high rates of new cases and deaths, and where the authorities face severe difficulties in meeting the health needs of its citizens.

This study aims to (a) measure the levels of fear of COVID-19, anxiety, stress, and depression of university students and any possible differences depending on gender and (b) test a model of structural equations with the possible variables related to depression, such as fear of COVID-19, stress, and anxiety. The hypotheses we studied were that: (1) women will show higher levels of fear of COVID-19, stress, anxiety, and depression than men; (2) fear of COVID-19 will have a positive relationship with depression, mediated through anxiety; and (3) stress will be positively and directly related to depression, also mediated through anxiety.

## Materials and Methods

### Participants

The population consisted of 78,059 students from four universities from the province of Manabí (Ecuador): Universidad Laica Eloy Alfaro de Manabí, Universidad Técnica del Litoral, Universidad Técnica de Manabí, and Universidad Estatal del Sur de Manabí. The sampling was incidental, due to the accessibility. A total of 640 undergraduates took part in the research. Of the full sample, 72% were women (*n* = 461) and 28% men (*n* = 179). The age of the participants ranged from 18 to 47 years (*M* = 21.69; *S.D* = 4.093).

### Instruments

We applied the Spanish version (Huarcaya-Victoria et al., 2020) of the FCV-19S (Ahorsu et al., 2020), which features a Likert-type scale made up of seven items [e.g., *My hands become clammy when I think about Coronavirus (COVID-19)*]. The instrument presented high reliability for the study sample (*α* = 0.904).

We also used the Spanish version (Fonseca-Pedrero et al., 2010) of the DASS-21 (Lovibond and Lovibond, 1995), which reports on stress levels (irritability, edginess, and/or inability to relax), anxiety (nervousness or physiological tension), and depression (feeling a loss of interest in daily activities, in life or in oneself) in the university population. This instrument is composed of three subscales, each containing seven items: (a) F1 = Stress (e.g., *I found it difficult to relax*), (b) F2 = Anxiety (e.g., *I was aware of dryness of my mouth*), and (c) F3 = Depression (e.g., *I could not seem to experience any positive feeling at all*). The instrument showed good reliability for the study sample (*α*^TOTAL^ = 0.954; *α*^STRESS^ = 0.907; *α*^ANXIETY^ = 0.861; and *α*^DEPRESSION^ = 0.875).

### Procedure

Before data collection, we first contacted the lecturers at several Ecuadorian universities who, under normal conditions, gave face-to-face lectures, but due to the COVID-19 lockdown, were currently teaching online. We organized a day on which the lecturers could respond to the online questionnaires. We also conferred with them *via* videoconferencing about how to complete the questionnaires and answered their queries.

On the day the questionnaires were collected, in Ecuador, 39,098 cases of sickness and 3,358 deaths from COVID-19 were recorded. At the end of the period for collecting the questionnaires, the number of patients was 84,370 and the number of deaths was 5,657 (Ministerio de Salud Pública del Ecuador, 2020; Servicio Nacional de Gestión de Riesgos y Emergencias, 2020).

Before the data were collected, all the participants, who were all over 18, gave their written informed consent. During the procedure, they were informed that no individual results, or any information that could identify them as study participants, would be published. Likewise, they were explicitly informed of the voluntary, anonymous, and confidential nature of the data provided and of the possibility of withdrawing at any time, without having to give any explanation or being penalized in any way. The questionnaires were completed individually and took approximately 15 min to complete.

The study was carried out in line with the ethical criteria established in the Declaration of Helsinki. The procedure was approved by the Ethical Committee of the Directorate for Research and Social and Technological Innovation of the University Laica Eloy Alfaro of Manabí (Ecuador), code II-COVID-19ULEAM2020.

### Data Analysis

Descriptive analyses with cut-off points were performed to analyze the mean scores on the DASS-21, and we took into account the cut-off points for the DASS-21 instrument given by other authors in order to obtain homologous samples of age, population, and culture. According to these authors, values of over 6 for stress and depression and 5 for anxiety showed the existence of mood disorders.

Comparative analyses were also conducted to establish differences between the scores for fear of COVID-19, stress, anxiety, and depression according to gender, for which the Mann-Whitney *U* test of independent samples was used. These analyses were carried out using the statistical program SPSS v.20. The confidence level applied in all the analyses was 95% (*p* < 0.05) or 99% (*p* < 0.01), depending on the case. The effect size was calculated with Cohen’s *d.*

A Structural Equation Model was performed to find out which variables are related to depression in undergraduate university students, using the EQS 6.2 program. Taking into account the ordinal nature of the data and the absence of normality, we decided to generate a polychoric correlation matrix (Flora and Curran, 2004) and use the robust estimation method (Bentler, 2006).

To evaluate the fit of the model, we took into account the values of the Satorra-Bentler chi-square (*x*^2^S-B), the Satorra-Bentler chi-square divided by degrees of freedom (*x*^2^S-B/*df*; values ≤2 were considered as optimal) and other indices which are not affected by the sample size: *Non-Normed Fit Index* (NNFI), *Normed Fit Index* (NFI), *Comparative Fit Index* (CFI), and *Incremental Fit Index* (IFI). As a criterion for assuming an adequate fit of the model, values of ≥0.95 in the above indices (Bentler, 1992) were established. For the *Root Mean Square Error of Approximation* (RMSEA), values between 0.05 and 0.08 were considered suitable to indicate an acceptable fit and ≤0.05 to indicate a good fit (Hu and Bentler, 1999).

## Results

### Fear of COVID-19, Stress, Anxiety, and Depression of University Undergraduates in Ecuador Based on Gender

The results of the Mann-Whitney *U* test of independent samples showed the existence of significant differences between men and women in the fear of COVID-19 scores (see Table 1). The scores obtained from the FCV-19S were higher for women than for men.

**Table 1:** Differences in fear of COVID-19, stress, anxiety, and depression scores according to gender.

Scale	Range (Men; *n* = 179)	Range (Women; *n* = 461)	*U*	*p*	*d*
Fear of COVID-19	255.84	345.61	29684.5	0.000^**^	0.45
Stress	304.8	326.6	38,449	0.180*^ns^*	0.10
Anxiety	304.51	326.71	38,397	0.171*^ns^*	0.10
Depression	315.34	322.5	40,336	0.659*^ns^*	0.05

*ns*, not significant; *d*, Cohen’s *d*.

** *p* < 0.01.

However, no statistically significant differences were detected for the levels of stress, anxiety, and depression between men and women studying in Ecuador.

### Structural Equation Model for Depression in University Undergraduates

The results obtained for the model of depression in university undergraduates during lockdown showed optimal goodness-of-fit indices: *χ*^2^S-B(341) = 894.2207, *p* < 0.001; *χ*^2^S-B/*df* stood at a value of ≤3, which is considered as optimal (Hu and Bentler, 1999), NFI = 0.99, NNFI = 0.98, CFI = 0.99, IFI = 0.99, and RMSEA = 0.05 [90% CI (0.046, 0.054)].

The model presented a good fit for men and women. For women, the model presented the following values: *χ*^2^S-B(341) = 808.2548, *p* < 0.001; NFI = 0.99, NNFI = 0.98, CFI = 0.99, IFI = 0.99, and RMSEA = 0.055 [90% CI (0.050, 0.059)], while for men, the model presented the following values: *χ*^2^S-B(342) = 610.7503, *p* < 0.001; NFI = 0.96, NNFI = 0.98, CFI = 0.98, IFI = 0.98, and RMSEA = 0.066 [90% CI (0.058, 0.075)].

**Figure 1:**
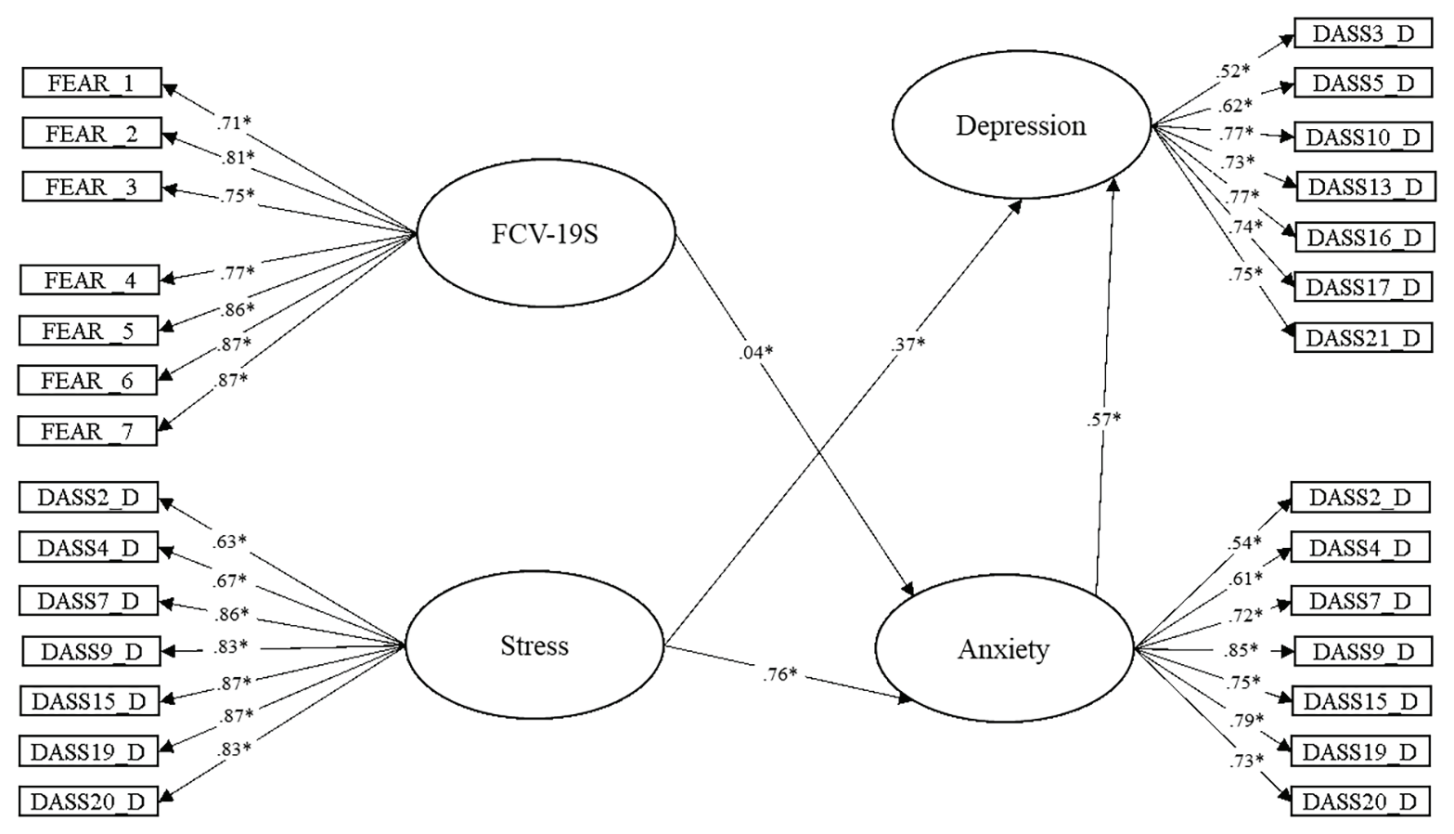
Significant relationships of the Structural Equation Modeling for the statistical prediction of depression in university undergraduates from Ecuador.

Figure 1 shows the Structural Equation Model for the relationship between fear of COVID-19 and stress in depression, with anxiety taken as a mediator of these variables. The relationship between fear of COVID-19 and depression is not shown in the figure as it is not significant. Due to the absence of normality, the robust maximum likelihood estimation method was used for the model (Mardia coefficient = 250.8931).

The model showed that the variables of fear of COVID-19 (*β* = −0.04; *p* < 0.05) and stress responses (*β* = 0.76; *p* < 0.05) have direct effects on anxiety during lockdown. The relationship between these variables accounts for 60.3% of the variance in anxiety.

The model also revealed that the variables for stress (*β* = 0.37; *p* < 0.05) and anxiety (*β* = 0.57; *p* < 0.05) reveal direct effects on depression during lockdown. The relationship between these model variables accounts for 79.8% of the variance in depression.

The standardized indirect effect values were: (a) for fear of COVID-19 > Anxiety > Depression = 0.023 and (b) for Stress > Anxiety > Depression = 0.43.

High, direct correlation values were observed in the polychoric matrix between the fear scale and the DASS-21 subscales (see Table 2). Similarly, the scores obtained show the existence of affective states of stress (*M* = 6.89), anxiety (*M* = 5.53), and depression (*M* = 5.93), as high scores were found in our study.

**Table 2:** Descriptive statistics and polychoric correlations for the Fear of COVID-19 Scale (FCV-19S) and subscales of the Depression and Anxiety Stress Scale (DASS-21).

	Scales	*n* (640)		1	2	3	4
		*M*	*S.D*				
1	Fear of COVID-19	14.37	5.381	1			
2	Stress	6.89	5.541	0.325	1		
3	Anxiety	5.53	4.989	0. 285	0.816	1	
4	Depression	5.93	5.077	0. 286	0.775	0.862	1

## Discussion

The primary objective of this research was to measure the levels of fear of COVID-19, anxiety, stress, and depression and their possible differences by gender among university students in Ecuador. It is clear that the participants, as a group, show high levels of stress, anxiety, and depression, and the levels recorded are above the cut-off points described in the scientific literature for each of these phenomena in similar populations of young undergraduates (e.g., Román et al., 2016). This shows that major psychological health problems exist among university students in the context of the COVID-19 lockdown, which is consistent with the moderate to severe symptoms of anxiety, stress, and depression observed in the Chinese population in the early stages of the pandemic (Huang and Zhao, 2020).

Our first hypothesis has been partially corroborated. Female undergraduates suffer higher levels of fear of COVID-19 than their male counterparts, as observed in the few similar studies carried out in other countries (Huang and Zhao, 2020; Sandín et al., 2020). However, no significant gender differences were found for levels of anxiety, stress, and depression, which does not concur with the observations of some studies carried out during the pandemic which show women to be more vulnerable to these disorders (e.g., Huang and Zhao, 2020; Sandín et al., 2020).

These findings provide new knowledge, since in most of the emergent scientific literature on this topic, these levels have been studied in health workers rather than undergraduates (Pappa et al., 2020). We suggest that the academic demands of university studies and the uncertain outlook take its toll on their learning and evaluation processes, leading to emotions of anxiety, stress, and depression in both female and male students, regardless of their gender.

The second main aim of the research was oriented toward studying how the influence of fear of COVID-19, stress, and anxiety relate to depression. We designed a model based on a review of the previous scientific literature and contrasted it with the collected data. The results obtained in the structural equations showed an excellent fit to the model. The model showed a good fit in both men and women. The second and third hypotheses of the study were, therefore, corroborated. The model shows how depression was directly and positively related by stress levels and indirectly through fear of COVID-19 and stress, mediated by the level of anxiety.

The relationship we observed for fear of COVID-19 with depression is consistent with some of the previous studies (Ahorsu et al., 2020; Alyami et al., 2020; Tzur Bitan et al., 2020). The relationships between fear of COVID-19 and anxiety (Mertens et al., 2020) and between anxiety and depression (Jansson-Fröjmark and Lindblom, 2008; Jacobson and Newman, 2017; Hovenkamp-Hermelink et al., 2019; Chen et al., 2020a) have also been documented separately. However, the relationship of fear of COVID-19 with depression, mediated by anxiety, has not been described before, making this a novel contribution. In addition, the relationship of stress with depression we observed concurs with some of the previous studies (Agoston and Rudolph, 2011; Slavich and Irwin, 2014; Park et al., 2019). The relationships between stress and anxiety and between anxiety and depression have been described previously (Díaz et al., 2012; Chen et al., 2020a). However, the influence of stress on depression, mediated by anxiety, during the social isolation caused by the pandemic, has not been described before in the literature. Furthermore, the indirect effect of FCV-19 on anxiety and depression was less than the indirect effect of stress on anxiety and depression.

The conditions in which people in Ecuador have to cope with the pandemic are extremely adverse in many ways. The rapid spread of the disease, the large number of people affected, the mounting number of deaths, a mistrust of the health system, ignorance, and disinformation may have all contributed significantly to the fact that young university students experience fear of COVID-19. This fear has been revealed as a factor that influences depression, and the effect of this fear on depression could be worsened by the existence of anxiety.

Undergraduate students tend to feel more fear when they feel they are in a more vulnerable situation and in greater danger, since many of them live away from the family home, and are unable to return and/or live in poor conditions where it is difficult to stay healthy and make ends meet.

Young female and male undergraduates are at a stage in their lives when they are planning their future, trying to find work, and trying to set up the conditions to become independent from their parents and fend for themselves. In the case of university students in Ecuador, most of them, even in normal conditions before the pandemic, were already making great sacrifices, together with their families, to meet the economic and academic requirements needed to be admitted to, follow, and make the most of a university education. Ecuador is a source country for emigration, due to the lack of job opportunities and its low standard of living and poor living conditions, which, due to the epidemic, are most likely to get worse.

The learning and assessment processes in university studies have been changed, in most cases, from face-to-face classes to distance and/or online learning, which has led to greater difficulties among students to access learning and adapt to the new methods. Many undergraduates formerly worked to earn some extra money but have been unable to continue, due to lockdown. All of these issues may well have increased stress levels, influencing depression. The stress produced by these long-lasting, dramatic changes faced by young university students can directly lead to symptoms of depression or initially result in a state of anxiety that could later lead to depression.

According to the results of this study, the uncertainty and the danger perceived by the undergraduates can become a fertile breeding ground for fear, stress, anxiety, and, as a result, depression. Knowing how complex the interactions are between these factors and worsening symptoms of depression, we urgently need to design intervention plans in universities to help these young people cope better with this type of situation.

The contributions of this study reinforce the observations made in a previous study that the university undergraduate population is more vulnerable, in a psycho-social sense, in the situation of pandemic and lockdown, than that of university graduates (Reznik et al., 2020). They also reinforce the conclusions drawn in some studies, which state that the lockdown measures have increased anxiety and depression among university undergraduates (Mazza et al., 2020; Santini et al., 2020). Our findings show that women, within this group, need preferential attention as regards strategies or measures to alleviate and prevent fear of COVID-19.

This study has certain limitations. The cross-sectional design, despite being suitable for the type of objectives we proposed, does not allow us to draw cause-effect conclusions. Future studies should be longitudinal to enable us to learn how the different factors affect the evolution of depression. The use of self-reports also has some limitations. The study was carried out in university students and the results cannot be generalized to the rest of the population. The sample is not balanced as regards gender, because it was incidental, which means that our interpretation of this variable is limited. The measurements used are all of the self-report type and subject to method variance effects and response biases, such as socially desirable responding. In future studies, this information-gathering technique should be supported by personal interviews. On the other hand, we consider that the instruments making up the self-report battery – which were previously validated and had good psychometric properties – were a success. In addition, another line of future research could study the relationship between the variables treated in this research and anxiety. Longitudinal studies could also be carried out to predict anxiety.

This study has allowed us to study in depth the complexity of depression in a situation of lockdown due to the pandemic. To continue progressing in future studies on the subject, it would be better to take into account registration measurements for other subject variables related to economic, employment status, housing conditions, goods and/or resources, among others. These sociodemographic aspects could shed more light on other factors which may be protectors or precursors of depression in a complex system which seems to be interwoven with fear, stress, and anxiety. It could also serve as a better to guide possible prevention and mitigation measures and optimize resources for the most vulnerable people within the overall group of university undergraduates.

Based on the model tested of depression in a pandemic, we propose different measures to prevent and alleviate this disorder. To reduce fear of COVID-19, it would be advisable to run convincing information campaigns about the disease, with training provided for its prevention and for effective coping strategies. Improving the health response could also help reduce fear, as there would be greater expectations of response in case of illness. Especially in the case of the university undergraduate population, the fear of COVID-19 could be increased by a feeling of inadequacy to face the difficult situation of the pandemic, by the lack of resources, to tackle a potential situation of disease and by poor or limited housing conditions, especially for undergraduate students living away from the family home.

Many of these university undergraduates have become ill and some have seen relatives become ill or die from COVID-19. Support plans with effective measures are needed to improve the undergraduates’ standard of living, eating habits, and living conditions. Many of them live in rooms or halls of residence in poor and/or overcrowded conditions. They should also be provided with internet access with sufficient bandwidth and the right hardware to be able to take part in distance learning. These steps could contribute to improving their ability to cope and/or to cushion the impact of these hardships.

The role of anxiety as a mediator between fear and stress and depression could lead to one innovative recommendation based on what we have observed in the present study. A program of attention and/or psychological training for undergraduates could be introduced, especially geared toward controlling and overcoming anxiety. Reducing anxiety could go a long way to alleviating the possible impact of fear and stress on depression. It would be advisable for the educational authorities, private bodies, and universities to urgently design and implement measures to alleviate these effects which harm the psychological health of their students. A society which does not protect and promote its young people’s health and development in the present puts its immediate future in jeopardy.

## Data Availability Statement

The raw data supporting the conclusions of this article will be made available by the authors, without undue reservation.

## Ethics Statement

The studies involving human participants were reviewed and approved by Ethical Committee of the Directorate for Research and Social and Technological Innovation of the University Laica Eloy Alfaro of Manabí (Ecuador). The patients/participants provided their written informed consent to participate in this study.

## Author Contributions

All authors made substantial contribution to the theoretical framework, design, data collection, or interpretation of this study. All authors contributed to this article and approved its publication.

### Conflict of Interest

The authors declare that the research was conducted in the absence of any commercial or financial relationships that could be construed as a potential conflict of interest.
